# Planning Capacity for Mental Health and Addiction Services in the Emergency Department: A Discrete-Event Simulation Approach

**DOI:** 10.1155/2019/8973515

**Published:** 2019-06-02

**Authors:** Deyvison T. Baia Medeiros, Shoshana Hahn-Goldberg, Dionne M. Aleman, Erin O'Connor

**Affiliations:** ^1^Mechanical and Industrial Engineering, University of Toronto, Toronto M5S 3G8, Canada; ^2^University Health Network, Toronto M5G 1J6, Canada

## Abstract

Ontario has shown an increasing number of emergency department (ED) visits, particularly for mental health and addiction (MHA) complaints. Given the current opioid crises Canada is facing and the legalization of recreational cannabis in October 2018, the number of MHA visits to the ED is expected to grow even further. In face of these events, we examine capacity planning alternatives for the ED of an academic hospital in Toronto. We first quantify the volume of ED visits the hospital has received in recent years (from 2012 to 2016) and use forecasting techniques to predict future ED demand for the hospital. We then employ a discrete-event simulation model to analyze the impacts of the following scenarios: (a) increasing overall demand to the ED, (b) increasing or decreasing number of ED visits due to substance abuse, and (c) adjusting resource capacity to address the forecasted demand. Key performance indicators used in this analysis are the overall ED length of stay (LOS) and the total number of patients treated in the Psychiatric Emergency Services Unit (PESU) as a percentage of the total number of MHA visits. Our results showed that if resource capacity is not adjusted, ED LOS will deteriorate considerably given the expected growth in demand; programs that aim to reduce the number of alcohol and/or opioid visits can greatly aid in reducing ED wait times; the legalization of recreational use of cannabis will have minimal impact, and increasing the number of PESU beds can provide great aid in reducing ED pressure.

## 1. Introduction

The rising number of emergency department (ED) visits in Ontario, Canada, is currently outpacing the population growth [1]. In seven years, the number of ED visits increased by 13.4% (from 5.2 million in 2008 to 5.9 million in 2014), while in the same period, the province's population only experienced a 6.2% growth [1]. According to Health Quality Ontario, visits to the ED are expected to rise even further, given that the population size in Ontario is estimated to grow by 30% in the next 25 years [1]. Additionally, there has also been an increase in ED visits related to mental health and addiction (MHA) in Ontario [2–8]. In particular, the number of MHA visits paid to the ED of Toronto Western Hospital (TWH), an academic hospital in Toronto, Ontario, increased by 55.7% in the last five years (2012–2016), compared to 14.5% for nonmental health and addiction (NMHA) visits. MHA visits account for 9.3% of all ED visits at TWH [5]. There is also evidence that ED visits for MHA have longer length of stay (LOS) compared to NMHA visits [5, 9, 10].

In response to prolonged wait times experienced in EDs and the impact of MHA on hospital use [11], there are system efforts underway to explore ways to improve the experience and impact of MHA ED visits. Several hospitals in the Greater Toronto Area have created separate MHA emergency units (usually within the ED) to provide streamlined care and safer environments for patients [12–15]. Another Ontario program to reduce the number of MHA ED visits is the META : PHI (Mentoring, Education, and Clinical Tools for Addiction : Primary Care-Hospital Integration), which provides evidence-based and timely treatment for patients with alcohol and opioid-related conditions through the use of Rapid Access Addiction Medicine (RAAM) clinics [16, 17]. Under the META : PHI care pathway, upon discharge from the ED, patients with alcohol and opioid-related dependence are referred to a RAAM clinic where they are seen by an addiction specialist within one week from their discharge date [18], thus contributing to a reduction in the number of addiction-related visits to the ED [19, 20]. At Health Sciences North in Sudbury, when comparing the number of times patients visited the ED 90 days before and after their first RAAM appointment, a 63% reduction in the number of ED visits was observed [19]. Similarly, in Sarnia, a 45% reduction has been reported [20]. As of July 2018, 29 RAAM clinics in 19 cities are listed on META : PHI's website (8 of which are located in Toronto) [21].

There are two major external factors to consider in the context of MHA visits to the ED, which may impact the already overburdened emergency system across Canada: (1) the current national opioid crisis [22] and (2) the legalization of recreational cannabis in October 2018 [23]. A 2017 report from the Canadian Institute for Health Information shows that the number of opioid overdose-related visits to the ED increased by almost 50% in the last five years (2012–2016) [8]. Additionally, evidence shows that legalization of the recreational use of cannabis can result in an increase of cannabis-induced visits to the ED [24–27]. In Colorado, cannabis-related ED visits increased by 19% in the first year after legalization in 2013 [28]. However, they also decreased by 27% in the following year (though it should be noted that this 27% only considers 9 months of comparable data, from Jan to Sept 2015) [28].

Provided that sustainable and efficient health care is based on adequate resource capacity to ensure that patients receive the care that they need in a timely manner [29], appropriate alignment between ED capacity and the growing demand should be sought. To this end, mathematical techniques, such as computer simulation, have been used for capacity planning and to improve the efficiency of healthcare services [29–36]. Most of these studies use DES and test scenarios of varying staff availability, scheduling adjustments, or changes to bed availability.

Drawing from the literature studies available, we initially quantify the number of substance abuse visits to TWH, which has a designated area within the ED for MHA cases, the PESU. We then use a discrete-event simulation (DES) to model ED capacity planning alternatives for the hospital, with special attention paid to potentially changing rates of MHA visits as a result of programs such as the META : PHI and the legalization of recreational cannabis in Canada. To the best of our knowledge, DES has not yet been applied in the context of modeling the impacts of MHA visits to the ED.

## 2. Methods

### 2.1. Emergency Department Overview

TWH has 272 beds [37] and is one of the four academic hospitals part of the University Health Network (UHN). TWH provides emergency care through its 34-bed ED and receives more than 60,000 visits a year [37]. In addition to these 34 beds, physically located within the ED, the Psychiatric Emergency Service Unit (PESU) has an additional four beds and two chairs, where up to six patients can stay at a time. The PESU is a safe and secure environment where MHA patients are directed for treatment upon arrival to the ED [12].

### 2.2. Patient Flow

**Figure 1:**
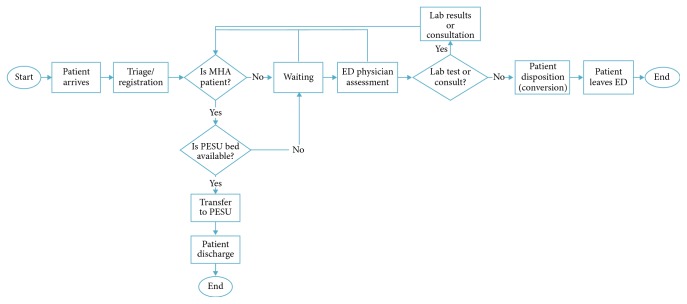
Patient flow in the ED/PESU [5].

Figure 1 depicts the process flow at TWH's ED. Patients arrive to the ED at TWH either on their own or by ambulance. Once in the ED, patients proceed to registration/triage, where they are seen by a registered nurse who assesses their health condition (e.g., reason for the visit, blood pressure, and body temperature) and provides them with an acuity score as per the Canadian Triage and Acuity Score (CTAS), which ranges from 1 to 5, with 1 being the most critical. After triage, if a stretcher is available, patients are generally assigned to a stretcher where they wait to see a physician. If a stretcher is not available, patients will remain in the waiting area until a stretcher is ready. When an ED physician initially sees a patient, lab or imaging tests may be ordered (e.g., blood work and ultrasound), and more complex cases may require the patient to be seen by another medical specialist (e.g., internal medicine and psychiatry). Patients who require testing and consultation must wait until their lab and imaging results are ready or until the consulting physician can see them. After a second assessment by the ED physician, patients commonly receive one of the following discharge dispositions: discharged, admitted, internal transfer, and external transfer. Discharged refers to patients who, after treatment, are sent to their place of residence; admitted refers to patients who were admitted to one of the hospital wards, operating room, or critical care unit; internal-transfer comprises all patients transferred within the hospital (e.g., to different clinics, or day surgery); and external transfers include all patients transferred to other acute or nonacute facilities outside of the hospital. Besides these 4 common dispositions, leaving against medical advice and dying during treatment are also possible dispositions.

For MHA patients, the flow through the ED is slightly different. After triage, if a bed is available in the PESU and the patient is medically stable and does not require a monitored bed, they are automatically transferred to the PESU. Otherwise, MHA patients occupy a regular bed in the ED until either a space in the PESU becomes available or until they are discharged (whichever comes first).

### 2.3. Data Source

This study was conducted using data collected from the National Ambulatory Care Reporting System (NACRS) and the Electronic Patient Records (EPR) system from UHN. The dataset included all visits paid to the TWH ED for a total of five fiscal years (i.e., 2012–2016), from April 2012 to March 2017. A fiscal year refers to the period from April 1^st^ to March 31^st^, the following year. Full approval from the Research Ethics Board was obtained, and all data used were anonymized and secured.

The International Statistical Classification of Diseases (ICD-10) associated with the main problem code recorded for each ED visit was used to group the data into two general categories: MHA and NMHA. The MHA group included the following ICDs: substance abuse (F55, F10–F19), schizophrenia (F20-excluding F20.4, F21–F25, F28–F29), mood and affective disorder (F30–F34, F39), anxiety/stress (F40–F45, F48), others—behavioral syndromes associated with physiological disturbances and physical factors, disorders of adult personality and behavior, and intentional self-harm (F50–F54, F59-F60, F63–F66, F68-F69, X60–X84). The NMHA group included all other ICDs.

Since this analysis pays special attention to substance abuse visits to the ED and to allow additional scenario testing (detailed in Section 2.6), the substance abuse group was further divided into 5 subgroups: alcohol (ICD F10), opioids (ICD F11), cannabis (ICD F12), multiple substance-related visits (ICD F19), which are visits in which two or more substances are combined, and visits for other reasons (ICDs F13–F18) including cocaine, tobacco, and hallucinogens.

### 2.4. Data Analysis

**Figure 2:**
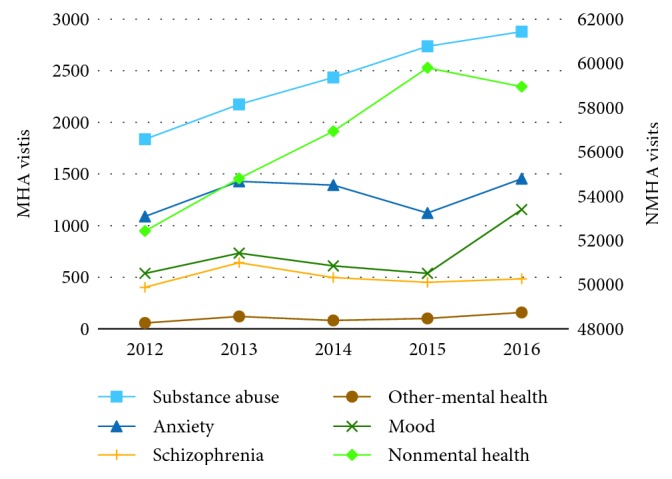
Behavior of ED visits over time by visit type.

Overall trends in the number of ED visits were calculated for both MHA and NMHA groups. To predict the future impacts of MHA ED visits, forecasting was used to estimate ED demand for fiscal years 2017 and 2018. Linear regression and Holt's method were used to forecast demand for trend data, and exponential smoothing, moving average, and weighted moving average were used for stationary data. The mean absolute deviation (MAD) was used to evaluate the performance of each forecasting method. The method leading to the lowest MAD was then used to produce the forecast. For NMHA, substance abuse, mood, anxiety, and other visits, which presented an upward linear trend, linear regression was employed. For schizophrenia cases which showed a stationary behavior, weighted moving average was used (Figure 2 and Table 1).

### 2.5. Simulation Model Development and Validation

The DES model of patient flow (Figure 1) was created using data from the fiscal year 2016 in Simul8, a discrete-event simulation software [38]. Stat:Fit [39] was used to fit statistical distributions to ED historical data. The model used current arrival rates for each visit type: NMHA, or one of the 5 MHA conditions—substance, schizophrenia, mood, anxiety, and other conditions. For processing times, the model also accounted for different acuity or CTAS (from 1 to 5) and discharge dispositions (i.e., discharged, admitted, internal and external transfer, and left/death upon arrival) associated with each visit type (Table 2).

To validate the simulation model, predicted and historical data were compared for the following parameters: (1) number of arrivals for both the NMHA and MHA group, (2) average ED LOS for both NMHA and MHA groups, and (3) ED LOS for NMHA and MHA combined average. ED LOS refers to the time from triage/registration to the time of discharge (i.e., in Figure 1, labeled as “Triage/Registration” to “Patient Discharge”). ED LOS is an important metric because for patients it represents the time they will be waiting to be serviced; from a quality-of-care perspective, several studies have linked prolonged ED LOS with adverse health outcomes [40–43]; also from a financial standpoint, the funding structure for hospitals in Ontario includes a pay for performance mechanism which awards financial incentives for hospitals that minimize ED LOS [44].

**Figure 3:**
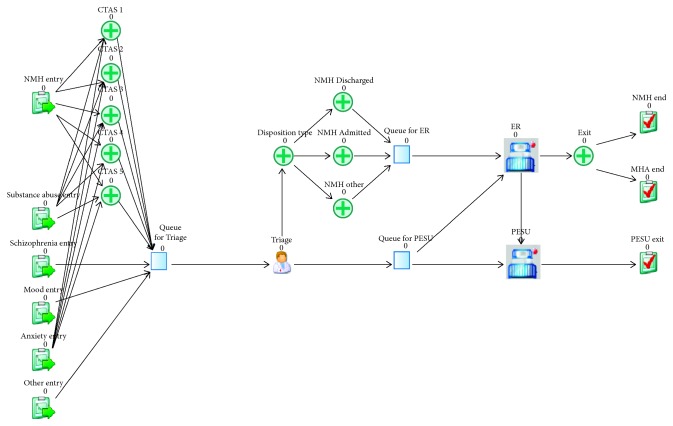
Screenshot from the simulation model.

In addition to comparing the outputs of the model with the original data sources, face validation was conducted by subject-matter experts at TWH to ensure accurate representation of the current system. All relevant metrics calculated from TWH's historical data fell with the 95% confidence interval of the simulation output (Table 3). Furthermore, the experts who reviewed the model were also satisfied with its performance. Hence, the model was deemed an appropriate representation of the current processes adopted in the hospital and was used to run further experiments. A screenshot of the simulation model can be seen in Figure 3.

### 2.6. Experimental Design

Five sets of experiments, based on specific motivating questions, were conducted to analyze different components of the system (Table 4). For succinctness, and because a previous study has already detailed the impacts on ED LOS when combining different factors (i.e., visit type-MHA and NMHA; CTAS; and disposition type) [5], the simulation results in this analysis will focus on two metrics: (1) the average overall ED LOS, which accounts for the ED LOS of both NMHA and MHA visits combined; (2) the number of MHA cases transferred to the PESU as a percentage of the total number of MHA arrivals to the ED. A more comprehensive list of metrics collected for each scenario tested is provided in Supplementary Materials Tables 5–10.

For each scenario, the simulation was run for 20 one-year (8,760 hours) replications. The warm-up period was 504 h (three weeks).

## 3. Results

### 3.1. MHA vs. NMHA: Frequency and ED LOS

**Figure 4:**
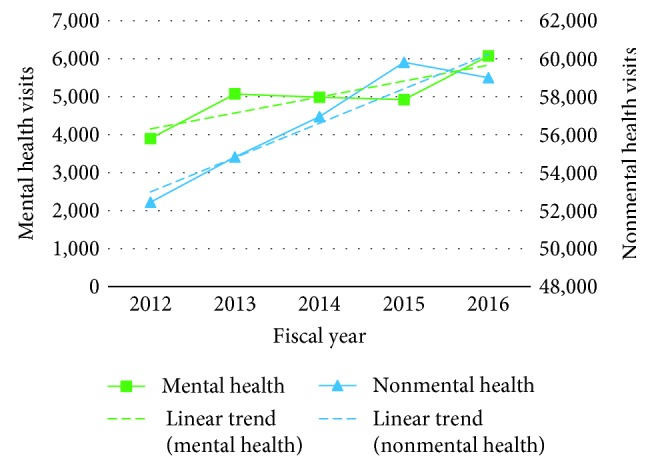
MHA ED visit frequency.

**Figure 5:**
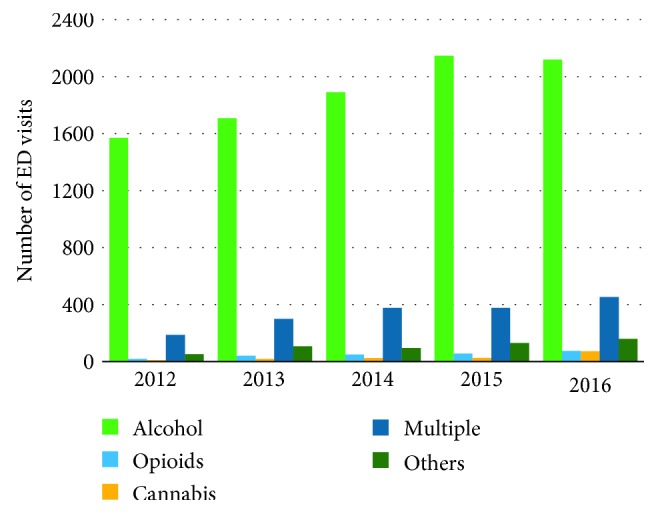
Frequency of substance abuse-related visits to the ED.

Between fiscal years 2012 and 2016, TWH received a total of 308,016 ED visits, out of which 25,114 visits (8.2%) were MHA visits. The total number of visits increased linearly by 15.5% from 56,345 to 65,078 (Figure 4). MHA visits rose from 3,923 to 6,131 visits (a 56.3% increase), while NMHA visits increased from 52,422 to 58,947 (a 12.4% increase). In 2015, there was a change in the Canadian Coding Standards for ICD-10-CA and the Canadian Classification of Health interventions, which may explain a small decrease in MHA visits for that specific year [46]. When considering substance-related ED visits only, all 5 subgroups also experienced linear: alcohol visits increased from 1,569 to 2,119 (35.0%), opioids from 19 to 74 (289.5%), cannabis from 10 to 73 (630.0%), multiple substances from 187 to 453 (142.3%), and other-cause visits from 52 to 160 visits (207.7%) (Figure 5). As of March 2017, alcohol visits accounted for 73.6% of all substance abuse visits, followed by 15.7% for multiple substances, and 5.6% for other visits. Opioid- and cannabis-related visits accounted for a small percentage of substance abuse visits 2.6% and 2.5%, respectively.

### 3.2. Forecasting MHA Visits to the ED

Given that each MHA subgroup presented in a different demand pattern (i.e., either trend or stationary series) (Figure 2) and that the simulation model differentiates the interarrival rate and length of stay for each MHA subgroup, the demand for each MHA patient subgroup and for NMHA was forecasted individually for fiscal years 2017 and 2018 (Table 1). Scenario A uses these forecasted values as inputs for the simulation model.

For scenario B, only part of the substance abuse group is considered (i.e., alcohol- and opioid-related visits). The 2016 base value for these cases combined (i.e., 2,193) is adjusted according to the parameters defined in Table 4. Similarly, for scenario C, the 2016 value for the number of cannabis visits (73) is adjusted according to the parameters defined in Table 4.

### 3.3. Simulation: Experimental Results

#### 3.3.1. Scenario A: ED LOS in 2017 and 2018

**Figure 6:**
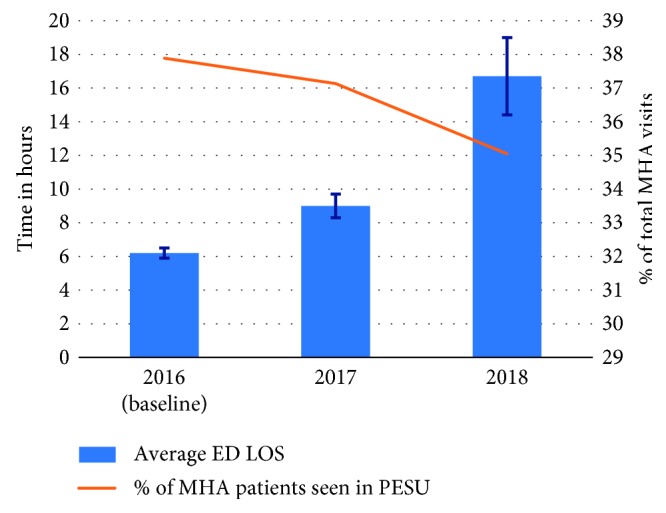
Scenario A: average ED LOS and percentage of MHA patients seen in PESU in 2018 and 2019.

Using the values forecasted for the years 2017 and 2018, the average overall ED LOS increased from 6.2 h at the baseline in 2016 to 9.0 h (51.6% increase) in 2017 and 16.7 h (169.4% increase) in 2018 (Figure 6). Also, since the PESU is operating close to its capacity, the number of MHA patients seen in the PESU as a percentage of the total number of MHA visits to ED only decreased from 37.9% at the baseline to 37.1% in 2017 and 35.0% in 2018. The numbers obtained in this scenario are intuitive, considering that if demand is expected to increase and no adjustments in capacity are made, the service level (e.g., wait times) is likely to deteriorate. The reader should also be mindful that, as depicted in Figure 1, whenever the PESU is operating at its capacity, the excess of MHA patients is treated in the regular ED.

#### 3.3.2. Scenarios B and C: Increase/Decrease in Substance Abuse Visits

**Figure 7:**
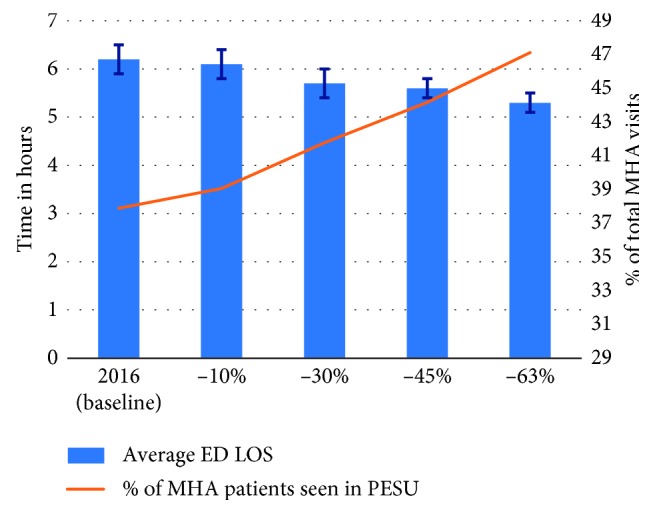
Scenario B: impact on average ED LOS and percentage of MHA patients seen in PESU given the decrease in demand for substance abuse.

In Scenario B, when reducing the arrival rate for alcohol- and opioid-related visits by 10%, 30%, 45%, and 63% the average overall ED LOS of 6.2 h at baseline decreased to 6.1 h, 5.7 h, 5.6 h, and 5.3 h, respectively (Figure 7). Moreover, when arrivals were reduced, the percentage of MHA patients seen in the PESU increased from 37.9% at the baseline to 39.1%, 41.8%, 44.2%, and 47.1%—note that the PESU capacity remains constant, so reducing the total number of MHA arrivals leads to an increment of the percentage of patients seen in the PESU. To exemplify this percent increase in patients serviced at PESU which may not be intuitive at first, consider that at most 6 patients can stay at the PESU at time (i.e., current scenario). Suppose that in the base scenario there were 30 MHA arrivals, the number of MHA cases transferred to the PESU as a percentage of the total number of MHA arrivals is 20% (i.e., 6/30); now suppose that MHA arrivals were reduced to 24, then the percentage of MHA patients seen in the PESU will increase to 25% (i.e., 6/24).

Results from Scenario B also highlight the benefits of implementing programs such as META : PHI, which include alleviating pressure from the ED/PESU by reducing the utilization of overburdened resources and likely improving patient care through reduction of wait times. Even if we consider demand reductions such as the ones observed in Sarnia (45%) and Sudbury (63%) to be overoptimistic for downtown Toronto, Scenario B shows that more conservative situations (i.e., with smaller reductions) can also produce a considerable impact on ED operations. For example, reducing the number of alcohol and opioid visits by 30%, which is equivalent to a 1% reduction in the overall ED volume, results in an 8.1% reduction in overall ED LOS (from 6.2 h to 5.7 h) and a 3.9% increase in MHA patients seen in the PESU (from 37.9% to 41.8%). Considering that most substance abuse patients are discharged from the hospital, the reduction in ED LOS can likely be attributed to the fact that discharged substance abuse visits are on average 1.8 h longer than discharged NMHA visits (5.8 h CI [5.6–6.0] versus 4.0 h CI [4.0–4.1]).

Another interesting result from Scenario B is the average MHA ED LOS (Table 6 in Supplementary Materials). Although one might have expected that reducing the number of alcohol- and opioid-related visits would produce a reduction in the average MHA ED LOS, the simulation results showed the opposite. When reducing the number of alcohol and opioid related visits by 10%, 30%, 45%, and 63%, the average MHA ED LOS increased from 7.7 h to 7.8 h, 7.9 h, 8.2 h, and 8.2 h, respectively (Table 6 in Supplementary Materials). These results are likely obtained because among MHA visits, the substance abuse group accounts for the largest population and it presents the second lowest average ED LOS, i.e., average 6.2 h and 95% CI [6.2–6.3], compared to the other MHA conditions, such as: schizophrenia, average 14.4 h and 95% CI [14.0–14.8]; mood disorders, average 12.1 h and 95% CI [11.4–12.7]; anxiety, average 4.0 h and 95% CI [3.9–4.1]; and other conditions, average 8.9 h and 95% CI [7.9–9.9]. In other words, removing a large portion of visits that account for one of the smallest average ED LOS from the MHA group, and keeping the visits that have a larger ED LOS, increased the overall average MHA ED LOS.

**Figure 8:**
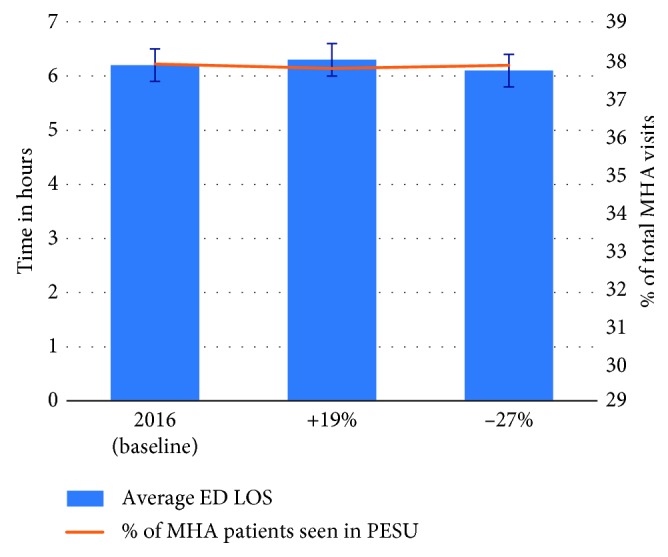
Scenario C: impact on average ED LOS and percentage of MHA patients seen in PESU given the increase in demand for substance abuse.

In Scenario C, when the arrival rate for cannabis-related visits at TWH was adjusted to mimic Colorado's experience with recreational cannabis legalization, the average overall ED LOS of 6.2 h at the baseline increased to 6.3 h for the first year and reduced to 6.1 h in the second year. Moreover, the number of MHA patients seen in the PESU slightly reduced from 37.9% at the baseline to 37.8% in the first year, and it increased again to 37.9% in the second year (Figure 8). These numbers show that—given the very small number of cannabis-related visits to the TWH's ED—the legalization will likely have almost no impact on ED flow.

#### 3.3.3. Scenario D: Increased PESU Capacity in 2016

**Figure 9:**
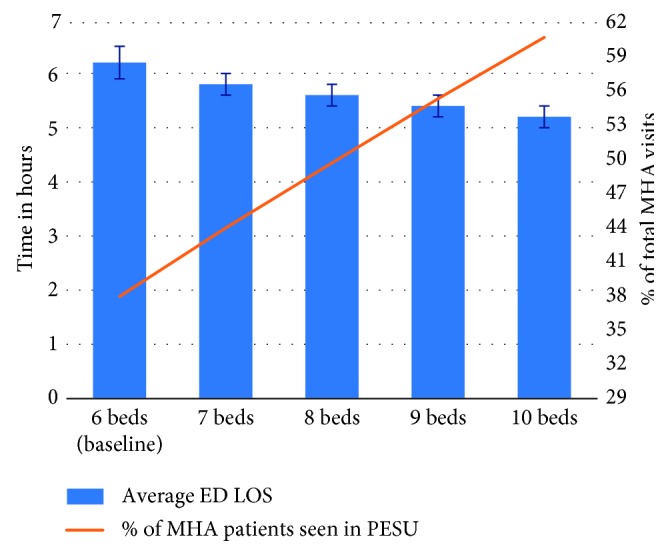
Scenario D: impact on average ED LOS and percentage of MHA patients seen in PESU given the increase in PESU bed capacity for 2016.

In Scenario D, when the number of beds in the PESU was increased from the current state of 6 beds to 7, 8, 9, or 10 beds, the overall average ED LOS reduced from the baseline of 6.2 h to 5.8 h, 5.6 h, 5.4 h, and 5.2 h, respectively (Figure 9). Furthermore, the percentage of MHA patients seen in the PESU increased from its current level of 37.9% to 43.8%, 49.5%, 55.1%, and 60.4% respectively. Note that, adding more beds to the PESU indeed led to a reduction in the overall ED LOS and a constant increase in the number of MHA seen in the PESU; however, the biggest reduction in ED LOS came from adding the first bed PESU (i.e., total of 7 beds) which reduced ED LOS by an average of 0.4 h, after that, each bed added only reduced ED LOS by an average of 0.2 h.

#### 3.3.4. Scenario E: Increased PESU Capacity in 2017 and 2018

**Figure 10:**
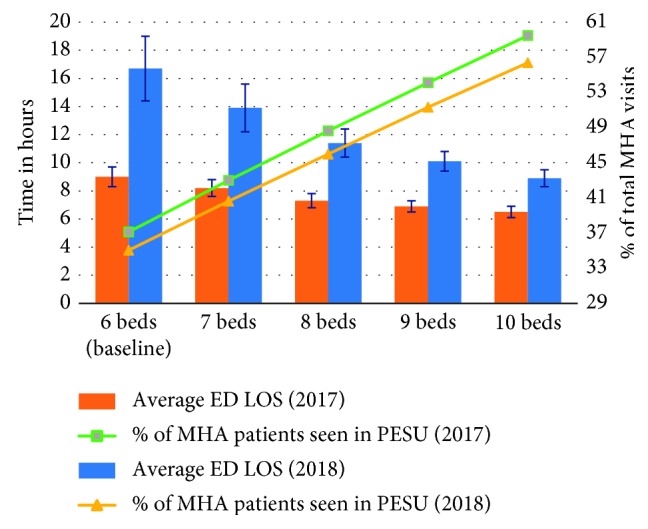
Scenario E: impact on average ED LOS and percentage of MHA patients seen in PESU given the increase in PESU bed capacity for both 2018 and 2019.

In Scenario E, when the number of PESU beds was increased following the numbers defined in Scenario D in combination with the demand forecasted for 2017 and 2018, the additional beds showed a positive and considerable impact on ED LOS as well as on the percentage of patients seen in the PESU (Figure 10). In 2017, increasing the number of beds to 7, 8,9, and 10 reduced the overall ED LOS from 9.0 h to 8.2 h, 7.3 h, 6.9 h, and 6.5 h, respectively, while the percentage of visits transferred to PESU increased from 37.1% to 43.0%, 48.6%, 54.1%, and 59.5%, respectively. Different from Scenario D, in which the biggest reduction in the overall ED LOS happened by adding one bed to the PESU, in this scenario—considering the demand for 2017—the largest reduction happens at 8 beds (i.e., with two additional PESU beds)). The initial bed added reduced the overall ED LOS by 0.8 h, the second bed by 0.9 h, and both the third and fourth bed by 0.4 h.

For 2018, increasing the number of beds to 7, 8, 9, and 10 reduced the overall ED LOS from 16.7 h to 13.9 h, 11.4 h, 10.1 h, and 8.9 h, while the percentage of visits transferred to PESU increased from 35.0% to 40.6%, 46.0%, 51.3%, and 56.4%, respectively (Figure 10). This scenario shows that the PESU capacity can greatly aid in reducing the overall ED LOS when demand further increases, in comparison with 2017, in which adding PESU beds led to an average ED LOS reduction of less than or equal to 0.9 h; in 2018, the smallest reduction is 1.2 h, but it goes up to 2.8 h when only one bed is added to the PESU (i.e., total of 7 beds).

## 4. Discussion

Given the current national opioid crisis and the legalization of recreational use of cannabis, this study quantified the number of substance abuse-related visits to a single academic hospital (i.e., TWH), this study also employed DES modeling to evaluate plausible scenarios that will likely impact ED wait times, such as demand fluctuations and resource capacity adjustments due to changes in the MHA landscape in Canada.

In congruency with other studies, our results showed that the overall number of MHA ED visits is increasing [3, 5–7], particularly in the number of substance abuse visits [2, 46, 47]. Through the use of demand forecasting models, we also found that the MHA demand for ED services is expected to grow even further.

After incorporating the forecasted demand into the DES models, we were able to quantify and show that the expected growth in demand will considerably impact LOS at TWH's ED. Prolonged LOS has been associated with poor patient management and clinical outcomes [40, 48–51], but specially for MHA patients—given the noisy and busy environment in the ED—longer waits potentially accentuate their psychological distress, which compromises their own safety and that of those around them (including other patients and staff) [5, 52–54], thus warranting possible ED process improvement and or/resource capacity adjustment (e.g., PESU number of beds).

Moreover, given the recent programs that have been spreading across the province to reduce the number of substance and opioid related visits to the ED by diverting these patients to alternative facilities and more specialized services [18, 55], we also used the DES model to evaluate the impact of such scenarios. Obtained results evidenced that patient flow in the ED could greatly benefit from care models such as the one stablished by the program META : PHI by diverting part of the substance abuse visits to other care settings, not only reduced the overall average ED LOS but also allowed the PESU, which is deemed a safer environment for patients in psychiatric crisis, to care for larger proportion of MHA patients who presented to the ED.

Even though we found that the legalization for recreational use of cannabis will likely not have a significant impact on TWH's ED resource utilization, two factors should be considered when interpreting these results: (1) TWH's ED number of cannabis visits could be misrepresented since data accuracy is often an issue when analyzing health administrative data, especially in substance abuse conditions when the type of substance may not be precisely recorded [56]; (2) our single-hospital results are not representative of other locations that have a well-documented history of struggling with substance abuse and addictions [57–63] (e.g., provinces such as British Columbia and Nova Scotia or the Thunder Bay district within Ontario). Stronger impacts on ED utilization may be observed in these places.

Lastly, we employed the DES model created to investigate how changes in PESU bed capacity could alleviate pressures from the ED. The results obtained showed that incrementing the number of PESU beds could meaningfully reduce ED LOS by servicing a larger percentage of MHA cases in the PESU. The benefits of adding more beds to the PESU became more evident when demand increase was also accounted in the model. Also, we were able to show what number of PESU beds could lead to the highest reduction in wait times.

Limitations of this study include the fact that the simulation model does not include hospital resources other than ED and PESU bed capacity. Much research is warranted to include additional staff involved in ED and PESU operations (including doctors, nurses, and social workers). Additionally, as with most simulation models, the model created here is specific to the site for which it was created, even though the overall patient flow observed in TWH's ED is similar to several other hospitals. Also, the lack of historical data from PESU LOS may compromise the validation process. Finally, because of the ICD coding mechanism, some cannabis, alcohol, and opioid visits may not have been accurately represented in the simulation model because they were hidden in the subgroup multiple substances.

## 5. Conclusion

Considering the increasing number of MHA visits to the ED, and the current opioid crisis Canada is facing combined with the legalization of recreational cannabis in October 2018, this study quantified the number of visits TWH received in five fiscal years (i.e., 2012–2016), used demand forecasting techniques to predict future ED demand, and employed a discrete event simulation model to evaluate the effects of demand and capacity adjustment in ED performance. The results obtained in this analysis showed that wait times are likely to deteriorate if no adjustment in resource capacity is made. We also found that programs such as META-PHI, which aim to reduce substance- and/or opioid-related conditions to the ED, can greatly contribute to reducing pressures from the ED. Moreover, cannabis legalization will have almost no impact on TWH's ED performance. Lastly, increasing PESU bed capacity will decrease overall ED wait times and increase the number of patients treated in a safer environment for people with MHA conditions.

## Figures and Tables

**Table 1:** Forecasted demand for fiscal years 2017 and 2018.

	Demand in 2016	Forecast in 2017 (% increase)^*∗*^	Forecast in 2018 (% increase)^*∗*^	Forecast method	Error (MAD)
NMHA	58,947	61,995 (5.2)	63,800 (8.2)	Linear regression	716.7
MHA (substance abuse)	2,879	3,206 (11.4)	3,471 (20.6)	Linear regression	43.4
MHA (schizophrenia)	484	486 (0.4)	486 (0.4)	Weighted moving average: *k* = 3 and weights (0.2, 8.0 and 0.8)	14.4
MHA (mood)	1,155	1,027 (−11.1)	1,131 (−2.1)	Linear regression	154.2
MHA (anxiety)	1,454	1,424 (−2.1)	1,467 (0.9)	Linear regression	136.6
MHA (others)	159	159 (0.0)	177 (11.3)	Linear regression	21.5

^*∗*^Compared to observed demand in 2017.

**Table 2:** Input parameters in the simulation model.

Parameter	Data/method	Details
Interarrival rates	Exponential distribution fit to historical ED arrival data	Interarrival rate distributions were separately created for the NMHA group and each MHA subgroup: substance abuse, schizophrenia, mood, anxiety, and others
Flow paths and proportions	Calculated from historical data	Proportions of patients in each acuity level (CTAS 1 to 5) were calculated. For each acuity level, proportions of each discharge disposition were also computed (i.e., discharged, admitted, internal transfer, external transfer, and left/death)
ED bed requirement	LogNormal distribution and average values fit to historical ED LOS data	Separate distributions were created for each acuity and discharge disposition combination
ED bed capacity	Supplied by ED manager	32 beds
PESU bed capacity	Supplied by PESU manager	6 beds
PESU LOS	Triangular distribution supplied by PESU manager	Minimal data were available, so estimates were used and validated by the PESU manager

**Table 3:** Numerical validation of the simulation model.

Metric	From historical data	Output from simulation (95% CI)
NMHA number of arrivals	58,947	58,939.3 (58,819.1–59,059.5)
Average NMHA ED LOS (h)	6.1	6.1 (5.8–6.3)
MHA number of arrivals	6,131	6,141.2 (6,108.5–6,174)
Average MHA ED LOS (h)	7.6	7.7 (7.6–7.8)
Average overall ED LOS (h)	6.2	6.2 (5.9–6.4)

**Table 4:** Scenario details.

Scenario ID	Question	Experimental details
A	What will happen in fiscal years 2017 and 2018 given the expected forecasted demand?	The interarrival rates for each patient group were adjusted to mimic the numbers obtained by demand forecasting (see Section 4.1)
B	What will happen if the number of alcohol- and opioid-related visits to the ED is reduced through new programs such as META : PHI?	Alcohol- and opioid-simulated interarrival rates were decreased randomly by 10% and 30%, as well as by 45% and 63% (similar to the numbers obtained by META : PHI program [19, 20])
C	What will happen if the number of cannabis-related visits to ED increases/decreases, following a similar pattern to other places after cannabis legalization?	To replicate Colorado's experience [28, 45], pretending that cannabis legalization happened in Canada at the end of 2016, cannabis-simulated interarrival rates were adjusted by +19% for 2017, and −27% for 2018.
D	What will happen if PESU bed capacity increases?	The number of beds in the PESU was increased from the initial 6 to 7, 8, 9, and 10 beds.
E	What will happen if PESU bed capacity is adjusted considering the expected growth in demand for 2017 and 2018?	The number of beds in the PESU was adjusted between 7 and 10 while changing interarrival rates to mimic the predicted demand in 2017 and 2018

## Data Availability

The historical data used to support the findings of this study may be released upon application to the University Health Network Research Ethics Board, who can be contacted at reb@uhnresearch.ca.

## Supplementary Materials

Supplementary Materials Additional metrics obtained from each simulated scenario are provided in the supplementary material (Appendix A, Tables 5–10).
