# Supplementary material for: Planning Capacity for Mental Health and Addiction Services in the Emergency Department: A Discrete-Event Simulation Approach
Source: J Healthc Eng. 2019 Jun 2;2019:8973515. doi: 10.1155/2019/8973515 (PMC6589296; doi:10.1155/2019/8973515)
Supplement: Supplementary Materials — Additional metrics obtained from each simulated scenario are provided in the supplementary material (Appendix A, Tables 5–10). [file 8973515.f1.pdf]

Supplementary Material

Additional metrics obtained from each simulated scenario are provided in supplementary material (Appendix A, Tables 5-10).

Appendix A: List of results for simulated scenarios

Table 5: Simulation results for Scenario A - Simulating forecasted demand for years 2017 and 2018

Metric	Base run 2016 (Avg. and 95% CI)	Forecasted 2017 (Avg. and 95% CI)	Forecasted 2018 (Avg. and 95% CI)
NMHA number of arrivals	58,939.3 (58,819.1 - 59,059.5)	61,960.7 (61,836.5 - 62,085.0)	63,799.2 (63,677.3 - 63,921.0)
Average NMHA ED LOS (h)	6.1 (5.8 - 6.3)	9.2 (8.4 - 9.8)	17.4 (14.9 - 19.9)
MHA number of arrivals	6,141.2 (6,108.5 - 6,174.0)	6,298.7 (6,266.4 - 6,1331)	6,749.8 (6,714.9 - 6,784.72)
Average MHA ED LOS (h)	7.7 (7.6 - 7.8)	7.7 (7.6 - 7.8)	7.7 (7.6 - 7.7)
Average overall ED LOS (h)	6.2 (5.9 - 6.4)	9.0 (8.3 - 9.7)	16.7 (14.4 - 19.1)
# patients seen in PESU	2,326.7 (2,320.1 - 2,333.20)	2,338.9 (2,331 - 2346.6)	2,365.6 (2,356.6 - 2,374.6)
ER occupancy (%)	88.8	93.3	95.2
PESU occupancy (%)	91.6	92.4	93.2

Table 6: Simulation results for Scenario B - Decrease in the number substance abuse visits (Alcohol and opioid visits related only)

**Metric**	**Base run 2017 (95% CI)**	**-10% (95% CI)**	**-30% (95% CI)**	**-45% (95% CI)**	**-63% (95% CI)**
Average NMHA ED LOS (h)	6.1 (5.8 - 6.3)	6.0 (5.7 – 6.4)	5.6 (5.4 – 5.8)	5.5 (5.2 – 5.7)	5.2 (5.0 – 5.4)
Substance abuse Arrivals	2,886.8 (2,865.1 - 2,908.4)	2,667.8 (2,647.2 – 2,688.4)	2,220.6 (2,200.6 – 2,240.6)	1,877.5 (1,861.4 – 1,893.6)	1,501.5 (1485.2 – 1,517.7)
MHA number of arrivals	6,141.2 (6,108.5 - 6,174.0)	5,922.3 (5,889.1 – 5,955.4)	5,475.1 (5,443.0 – 5,507.1)	5,132.0 (5,103.7 – 5,160.2)	4,755.9 (4,729.4 – 4,782.4)
Average MHA ED LOS (h)	7.7 (7.6 - 7.8)	7.8 (7.7 – 7.8)	7.9 (7.8 – 8.1)	8.2 (8.1 – 8.3)	8.2 (8.0 – 8.3)
Average overall ED LOS (h)	6.2 (5.9 - 6.4)	6.1 (5.8 – 6.4)	5.7 (5.5 – 5.9)	5.6 (5.4 – 5.8)	5.3 (5.2 – 5.5)
# patients seen in PESU	2,326.7 (2,320.1 - 2,333.2)	2,312.9 (2,307.0 – 2,318.7)	2,287.5 (2,280.5 – 2,294.5)	2,266.9 (2,259.8 – 2,274.0)	2,240.8 (2,234.0 – 2,247.5)
ER occupancy (%)	88.8	87.3	86.5	87.4	86.6
PESU occupancy (%)	91.6	90.8	89.8	89.4	88.0

Table 7: Simulation results for Scenario C – Increase and decrease in the number substance abuse (Increase and decrease in the number of cannabis related visits only)

Metric	Base run 2017 (95% CI)	+19% (95% CI)	-27% (95% CI)
Average NMHA ED LOS (h)	6.1 (5.8 - 6.3)	6.2 (6.0 – 6.5)	6.0 (5.7 – 6.4)
Substance abuse Arrivals	2,886.8 (2,865.1 - 2,908.4)	2,906.1 (2,884.4 – 2,927.7)	2,896.0 (2,874.6 – 2,917.3)
MHA number of arrivals	6,141.2 (6,108.5 - 6,174.0)	6,160.5 (6,127.7 – 6,193.3)	6,150.4 (6,117.8 – 6,183.0)
Average MHA ED LOS (h)	7.7 (7.6 - 7.8)	7.7 (7.6 – 7.9)	7.8 (7.7 – 7.8)
Average overall ED LOS (h)	6.2 (5.9 - 6.4)	6.3 (6.1 – 6.6)	6.1 (5.8 – 6.4)
# patients seen in PESU	2,326.7 (2,320.1 - 2,333.2)	2,326.9 (2,320.1 – 2,333.7)	2,328.2 (2,320.9 – 2,335.4)
ER occupancy (%)	88.8	89.4	89.1
PESU occupancy (%)	91.6	91.4	91.6

Table 8: Simulation results for Scenario D - Change in capacity - number of PESU BEDS

Metric	Base run 2016 (6 beds) (95% CI)	2016 (7 beds) (95% CI)	2016 (8 beds) (95% CI)	2016 (9 beds) (95% CI)	2016 (10 beds) (95% CI)
Average NMHA ED LOS (h)	6.1 (5.8 - 6.3)	5.7 (5.5 - 6.0)	5.5 (5.3 - 5.7)	5.3 (5.1 – 5.5)	5.1 (4.9 – 5.2)
Average MHA ED LOS (h)	7.7 (7.6 - 7.8)	7.7 (7.6 - 7.8)	7.7 (7.6 - 7.8)	7.7 (7.6 – 7.8)	7.8 (7.7 – 7.9)
Average overall ED LOS (h)	6.2 (5.9 - 6.4)	5.8 (5.6 - 6.1)	5.6 (5.4 - 5.8)	5.4 (5.2 – 5.6)	5.2 (5.0 – 5.3)
# patients seen in PESU	2,326.7 (2,320.1 - 2,333.20)	2,692.3 (2,685.0 - 2,699.5)	3,040.4 (3,032.9 – 3,047.9)	3,382.5 (3,373.5 – 3,391.5)	3,710.5 (3,698.2 – 3,722.8)
ER occupancy (%)	88.8	87.7	86.8	86.2	85.4
PESU occupancy (%)	91.6	90.5	89.9	88.2	87.7

Table 9: Simulation results for Scenario E Part 1- Combined change in demand and PESU bed capacity (2017)

Metric	2017 (6 beds) (95% CI)	2017 (7 beds) (95% CI)	2017 (8 beds) (95% CI)	2017 (9 beds) (95% CI)	2017 (10 beds) (95% CI)
Average NMHA ED LOS (h)	9.2 (8.4 - 9.8)	8.3 (7.6 - 8.9)	7.3 (6.8 - 7.8)	6.9 (6.5 – 7.3)	6.4 (6.1 – 6.8)
Average MHA ED LOS (h)	7.7 (7.6 -7.8)	7.7 (7.6 - 7.8)	7.6 (7.5 - 7.7)	7.6 (7.5 – 7.7)	7.7 (7.6 – 7.8)
Average overall ED LOS (h)	9.0 (8.3 - 9.7)	8.2 (7.7 - 8.8)	7.3 (6.9 - 7.8)	6.9 (6.5 – 7.3)	6.5 (6.1 – 6.8)
# patients seen in PESU	2,338.9 (2,331.0 – 2,346.6)	2,707.7 (2,699.1 - 2,716.3)	3,062.7 (3,055.9 - 3,069.4)	3,408.5 (3,397.8 – 3,419.2)	3,746.1 (3,733.9 – 3,758.2)
ER occupancy (%)	93.3	92.3	91.2	90.7	90.0
PESU Occupancy (%)	92.4	91.4	90.9	89.7	88.5

Table 10: Simulation results for Scenario E Part 2 - Combined change in demand and PESU bed capacity (2018)

Metric	2018 (6 beds) (95% CI)	2018 (7 beds) (95% CI)	2018 (8 beds) (95% CI)	2018 (9 beds) (95% CI)	2018 (10 beds) (95% CI)
Average NMHA ED LOS (h)	17.4 (14.9 - 19.9)	14.3 (12.5 - 16.0)	11.6 (10.5 - 12.7)	10.2 (9.4 – 11.0)	9.0 (8.3 – 9.6)
Average MHA ED LOS (h)	7.7 (7.6 - 7.7)	7.7 (7.6 - 7.8)	7.6 (7.5 - 7.7)	7.7 (7.6 – 7.8)	7.7 (7.5 – 7.8)
Average overall ED LOS (h)	16.7 (14.4 - 19.1)	13.9 (12.2 - 15.5)	11.4 (10.4 - 12.5)	10.1 (9.3 – 10.8)	8.9 (8.3 – 9.5)
# patients seen in PESU	2,365.6 (2,356.6 - 2,374.6)	2,742.8 (2,735.2 - 2,750.4)	3,104.4 (3,095.6 - 3,113.1)	3,463.5 (3,453.3 – 3,473.6)	3,805.6 (3,792.7 – 3,818,5)
ER occupancy (%)	95.2	94.4	98.1	93.2	93.1
PESU Occupancy (%)	93.2	92.5	92.0	90.6	90.2
